# Effectiveness of LGBTQ targeted cognitive behavioral therapy on mental health outcomes: A systematic review and meta-analysis protocol

**DOI:** 10.1371/journal.pone.0359815

**Published:** 2026-11-10

**Authors:** J. Doris Chi, Yi Yang, Shi-Yi Wang, Kate Nyhan, Skyler D. Jackson

**Affiliations:** 1 Department of Social and Behavioral Sciences, Yale School of Public Health, New Haven, Connecticut, United States of America; 2 Department of Psychiatry and Behavioral Sciences, Emory University School of Medicine, Atlanta, GeorgiaUnited States of America; 3 Department of Chronic Disease Epidemiology, Yale School of Public Health, New Haven, Connecticut, United States of America; 4 Harvey Cushing/John Hay Whitney Medical Library, Yale University, New Haven, Connecticut, United States of America; 5 Department of Environmental Health Sciences, Yale School of Public Health, New Haven, Connecticut, United States of America; Brown University, UNITED STATES OF AMERICA

## Abstract

**Introduction:**

Sexual and gender minority populations experience disproportionately high rates of mental disorders due, in large part, to their chronic exposure to stigma, discrimination, and minority stress. Cognitive behavioral therapy (CBT) is an established evidence-based psychotherapy; however, conventional CBT was primarily developed among predominantly cisgender, heterosexual individuals and not often addresses identity-specific stressors relevant to LGBTQ individuals. Emerging adaptations of LGBTQ-affirmative CBT integrate components that affirm sexual and gender minority identities and target minority stress. Although the number of investigations testing such interventions has increased, evidence remains fragmented across studies. To our knowledge, no systematic review has examined the efficacy of LGBTQ targeted CBT on improving mental health outcomes.

**Objective:**

This systematic review aims to synthesize empirical evidence from randomized controlled trials (RCTs) evaluating the effectiveness of LGBTQ targeted CBT interventions in reducing mental health symptoms among sexual and gender minority adults.

**Materials and methods:**

A comprehensive search will be conducted across PubMed, EMBASE, PsycINFO, the Cochrane Library, and Scopus, supplemented by gray-literature searches. Eligible studies include RCTs conducted on adults aged 18–65 and evaluating LGBTQ targeted CBT compared with usual care, waitlist, or active controls. Outcomes include depression, anxiety, obsessive-compulsive disorder, post-traumatic stress disorder, and substance use disorder symptom changes at post-intervention and follow-up. Data extraction and risk-of-bias assessment will be performed independently by two reviewers, with risk of bias assessed using the Cochrane Risk of Bias 2 tool. Where feasible, meta-analyses using random-effects models in R will estimate standardized mean differences or risk ratios with 95% confidence intervals. Overall efficacy and potential moderators, such as intervention delivery mode and duration, will be investigated.

**Expected contribution:**

By consolidating the evidence base for LGBTQ targeted CBT, this review will clarify its efficacy, identify key intervention features associated with improved outcomes, and provide guidance for evidence-based mental health care for sexual and gender minority populations.

**PROSPERO registration:**

This protocol has been pre-registered with the International Prospective Register of Systematic Reviews (PROSPERO: CRD420251173207).

## Introduction

### LGBTQ mental health

The lesbian, gay, bisexual, transgender, and queer (LGBTQ) population encompasses a wide range of identities and experiences around the world. Despite this diversity, a consistent pattern emerges across cultural settings: sexual minority individuals often remain socially marginalized and invisible, facing numerous risk factors that threaten their physical and psychological well-being [1]. Research continues to show that compared to heterosexual individuals, gender and sexual minorities are disproportionately affected by mental health challenges, such as higher rates of depression, anxiety, and substance misuse [2–5].

A widely cited explanation for these disparities is the minority stress model [6,7], which argues that chronic exposure to stigma-related stressors, including discrimination, victimization, and harassment, contributes significantly to negative health outcomes. Expanding on this perspective, the psychological mediation framework [8] emphasizes that persistent exposure to prejudice and social exclusion disrupts both internal processes (e.g., self-regulation, coping) and interpersonal relationships, ultimately increasing vulnerability to mental health disorders. Given that minority stress influences cognitive, emotional, and behavioral functioning [9], it is closely linked to mental health difficulties as well as various health risk behaviors. Depression, anxiety disorders, post-traumatic stress disorder (PTSD), obsessive-compulsive disorder (OCD), and substance use disorders represent some of the most prevalent and impairing mental health conditions among LGBTQ individuals and are strongly associated with minority stress exposure. With the ongoing disproportionate mental health burden faced by LGBTQ individuals, it is crucial to address these disparities by identifying and implementing effective, evidence-based interventions that can reduce their mental health symptoms and promote well-being.

### Prior evidence on CBT for LGBTQ populations

Psychotherapy is well established as an effective treatment for a wide range of mental health problems. Among these approaches, cognitive behavioral therapy (CBT) is one of the most empirically supported treatments and is suitable for addressing cognitive, affective, and behavioral minority stress processes [10]. However, traditional CBT protocols were largely developed and validated in cisgender, heterosexual samples and were not specifically designed to address minority stress processes or identity-specific challenges, such as stigma, concealment, or rejection, that are particularly salient for LGBTQ individuals [11].

In response, researchers have developed LGBTQ-affirmative CBT adaptations that integrate strategies to target minority stress-related cognitive, affective, and behavioral mechanisms. For instance, a 10-session transdiagnostic CBT intervention targeting young sexual minority men produced small-to-moderate improvements in depression, anxiety, substance use, and suicidality, highlighting the potential of minority stress-focused CBT to address co-occurring mental health difficulties [12]. Another trial with gender-diverse sexual minority women found that affirmative CBT produced large reductions in depression and anxiety relative to a waitlist control [13]. Two randomized controlled trials (RCTs) of LGBTQ-affirmative internet-based CBT, conducted among LGBTQ youth in the United States [14] and sexual minority men in China [15], found that the interventions reduced psychological distress and improved depression and anxiety symptoms, with stronger effects among participants exposed to higher levels of stigma.

Together, these findings indicate that CBT, when affirmatively adapted, might yield meaningful improvements in mental health outcomes for LGBTQ populations. These outcomes, including depression, anxiety, PTSD, OCD, and substance use disorders, are also among those with the most robust empirical support for CBT, making them theoretically and clinically appropriate targets for evaluating the effectiveness of LGBTQ adapted CBT interventions. Nonetheless, the evidence base remains scattered. Many trials involve small samples, brief follow-ups, and heterogeneous outcome measures that limit comparability across studies. This highlights the need for systematic synthesis to clarify the overall effectiveness of LGBTQ targeted CBT interventions.

### Gaps in the literature

Although the number of RCTs on LGBTQ-affirmative CBT has grown, few reviews have synthesized evidence from these trials across mental health outcomes. A recent systematic review [16] examined evidence-based cognitive and/or behavioral interventions for LGBTQ populations, but it did not restrict inclusion to interventions explicitly grounded in CBT or evaluate trials that integrated both cognitive and behavioral components. A CBT-specific synthesis is therefore necessary to reduce conceptual heterogeneity, enable more accurate estimation of CBT-related outcomes, and clarify whether the theorized synergistic effects of jointly applied cognitive and behavioral components are empirically supported. In addition, Dunn et al. [17] conducted a preliminary meta-analysis examining CBT for internalizing disorders among LGBTQ populations and reported promising effects. However, the review did not distinguish LGBTQ adapted CBT from standard CBT and included non-controlled trials, increasing the risk of bias. Consequently, it remains unclear whether LGBTQ-affirmative CBT, as a distinct intervention, demonstrates consistent efficacy across mental health domains. Furthermore, the above two studies did not include sexual health or substance use outcomes within the broader scope of mental health. The present review addresses these limitations by focusing specifically on RCTs of LGBTQ adapted CBT interventions and by including studies in which sexual health- or substance use-related interventions incorporate mental health outcomes alongside behavioral ones.

This systematic review will comprehensively identify and synthesize evidence from RCTs of LGBTQ targeted CBT for adults aged 18–65, encompassing mental health outcomes such as depression, anxiety, OCD, PTSD, and substance use disorders. By summarizing effect sizes and exploring moderators such as delivery mode and intervention duration, the review aims to provide a clearer, evidence-based foundation for responsive clinical practice and future research.

## Methods and analysis

### Research objectives

This systematic review will address the question: “What is the effectiveness of LGBTQ targeted CBT in improving mental health outcomes such as depression, anxiety, OCD, PTSD, and substance use disorders among sexual and gender minority populations?” The question was formulated using the adapted PICO framework. The review aims to synthesize empirical evidence on LGBTQ targeted CBT and to inform guidelines for clinicians in improving mental health care for sexual and gender minority populations.

### Study pre-registration

This systematic review protocol was prepared in accordance with the Preferred Reporting Items for Systematic Review and Meta-Analyses Protocols (PRISMA-P) guidelines [18] (S1 Appendix). It has been pre-registered with the International Prospective Register of Systematic Reviews (PROSPERO; CRD420251173207, registered on October 27, 2025). The document outlines the eligibility criteria, search strategy, data extraction process, and risk of bias assessment to promote transparency, reproducibility, and methodological rigor throughout the review.

### Inclusion criteria

**Types of studies.** We will include only RCTs that evaluate LGBTQ targeted CBT interventions. No restrictions will be placed on publication year, geographic location, or specific affirmative adaptations, such as integration of minority stress psychoeducation, identity-affirming cognitive restructuring, and stigma coping skills.

**Types of participants.** Eligible studies will include adults aged 18–65 years who identify as lesbian, gay, bisexual, transgender, queer, or with other sexual and gender minority identities.

**Types of intervention.** Interventions must explicitly target LGBTQ populations (i.e., include adaptations or modifications targeting LGBTQ populations) and be based on CBT principles. Eligible formats include in-person, videoconference, telephone, or live-chat modalities, delivered in any setting, including outpatient and inpatient facilities, charitable organizations, educational institutions, or any other community or home settings.

**Types of comparators.** Comparator conditions may include treatment as usual, waitlist, or active control interventions.

**Types of outcomes.** Eligible studies must assess mental health-related outcomes, including but not limited to depression, anxiety, OCD, PTSD, or substance use disorders. Both immediate post-intervention and follow-up outcomes will be included.

### Exclusion criteria

The exclusion criteria include:

1. Studies focusing on children and adolescents (<18 years) or older adults (>65 years) will be excluded; studies with a mixed sample age range will be assessed on a case-by-case basis.
2. Studies including participants who presented with current active suicidality or homicidality (excluding passive suicidality), psychotic symptoms, or significant cognitive impairment.
3. Studies involving participants who were undergoing two or more concurrent therapies during enrollment in the RCT.
4. Reviews, observational studies, case reports, abstracts, cohort studies, lab-based experimental studies, single-arm studies, and other types of research studies.
5. Non-CBT interventions.
6. Studies that only examined behavioral outcomes, such as HIV risk reduction, substance use frequencies, or sexual behaviors.
7. Studies with th incomplete data or insufficient information to determine intervention outcomes.

The use of concurrent psychotropic medication will not constitute an exclusion criterion. Information regarding medication status, such as the proportion of participants receiving medication, will be extracted and considered in sensitivity or subgroup analyses where feasible.

### Information sources and search strategy

An initial search strategy for RCTs was developed and pilot-tested on Ovid MEDLINE on October 26, 2025, to evaluate keywords and indexing terms relevant to LGBTQ  targeted CBT interventions. Following this preliminary testing, and with input from a public health librarian, the finalized search strategy will be adapted for each database. The following electronic databases will be searched: PubMed, EMBASE (Ovid), PsycINFO (Ovid), the Cochrane Library, and Scopus. To capture additional relevant materials, we will search for trial registrations through the ClinicalTrials.gov database. We will conduct both forward and backward citation searching to identify additional eligible studies [19]. The search strategy will be implemented by one reviewer (K.N.). The preliminary search terms on Ovid MEDLINE are provided in Table 1. The finalized formal search will be conducted as planned on PubMed, EMBASE (Ovid), PsycINFO (Ovid), Cochrane Library, and Scopus.

**Table 1:** Search terms on MEDLINE (Ovid).

#	Query	Results from 4 Dec 2025
1	(gender* adj2 nonconform*).mp.	908
2	(gender* adj2 non-conform*).mp.supp	368
3	(trans adj (female* or male* or man or men or women or woman or boy* or girl*)).mp.	1,049
4	(trans adj (population* or patient* or participant* or subject* or adolescent* or teen* or child* or individual* or people or person* or youth*)).mp.	1,006
5	agender*.mp.	66
6	bicurious.mp.	4
7	bigender*.mp.	8
8	bisexual*.mp.	16,210
9	cross sex.mp.	665
10	crossgender.mp.	2
11	DSD.mp.	3,377
12	gay.mp.	17,279
13	gays.mp.	598
14	gender change.mp.	99
15	gender crossing.mp.	4
16	gender dysphori*.mp.	2,907
17	gender fluid*.mp.	97
18	gender identit*.mp.	27,909
19	gender incongruen*.mp.	558
20	gender minorit*.mp.	17,050
21	gender neutral.mp.	859
22	gender queer.mp.	46
23	gender transition*.mp.	392
24	gender varian*.mp.	215
25	genderless.mp.	20
26	genderqueer*.mp.	228
27	GLB.mp.	661
28	GLBQ.mp.	3
29	GLBs.mp.	25
30	GLBT.mp.	146
31	GLBTQ.mp.	15
32	heteroflexible.mp.	6
33	homosexual*.mp.	43,774
34	intersex*.mp.	4,284
35	lesbian*.mp.	10,811
36	lesbigay*.mp.	5
37	LGB.mp.	1,674
38	LGBQ.mp.	303
39	LGBS.mp.	92
40	LGBT*.mp.	7,774
41	men who have sex with men.mp.	17,906
42	mostly-heterosexual.mp.	166
43	MSM.mp.	15,755
44	MSMW.mp.	152
45	nonbinary.mp.	1,776
46	non-binary.mp.	1,734
47	nonheterosexual*.mp.	234
48	non-heterosexual*.mp.	580
49	queer.mp.	3,931
50	queers.mp.	50
51	same gender loving.mp.	37
52	same sex couple*.mp.	527
53	same sex relations*.mp.	448
54	same-sex attract*.mp.	390
55	sexual identit*.mp.	3,051
56	sexual minorit*.mp.	6,456
57	sexual orientation*.mp.	10,489
58	sexual preference*.mp.	808
59	SGM.mp.	1,714
60	third gender*.mp.	88
61	transboy*.mp.	9
62	transex*.mp.	213
63	transfeminine.mp.	405
64	transgender*.mp.	18,406
65	transgirl*.mp.	13
66	transm#n.mp.	499
67	transmasculine.mp.	532
68	transsex*.mp.	5,633
69	trans-sex*.mp.	93
70	trans-spectrum.mp.	9
71	transwom#n.mp.	393
72	two-spirit*.mp.	262
73	women loving women.mp.	9
74	women who have sex with women.mp.	227
75	WSW.mp.	225
76	WSWM.mp.	26
77	exp “sexual and gender minorities”/	23,039
78	bisexuality/	5,232
79	transsexualism/	4,816
80	exp homosexuality/	37,562
81	gender identity/	23,642
82	health services for transgender persons/	224
83	gender dysphoria/	1,217
84	exp “Disorders of Sex Development”/	37,273
85	[this chunk of terms comes from this paper: Abboud, S., Veldhuis, C., Ballout, S., Nadeem, F., Nyhan, K., & Hughes, T. (2022). Sexual and gender minority health in the Middle East and North Africa Region: A scoping review. International Journal of Nursing Studies Advances, 4, 100085. https://doi.org/10.1016/j.ijnsa.2022.100085]	0
86	or/1–85	151,392
87	exp Cognitive Behavioral Therapy/	42,646
88	(cbt or cognitive behavio* therapy).mp.	49,822
89	87 or 88	58,156
90	(randomized controlled trial or controlled clinical trial).pt. or randomized.ab. or placebo.ab. or clinical trials as topic.sh. or randomly.ab. or trial.ti.	1,734,958
91	exp animals/ not humans.sh.	5,402,135
92	90 not 91	1,600,970
93	[that’s the Cochrane Highly Sensitive Search Strategy for identifying randomized trials in MEDLINE: sensitivity- and precision-maximizing version (2008 revision)]	0
94	86 and 89 and 92	126
95	[for comparison: here’s a Cochrane search strategy for CBT]	0
96	COGNITIVE THERAPY/ or BEHAVIOR THERAPY/ or (cogniti$ adj3 (behavio$ or intervention$ or psychotherap$ or technique$ or therap$ or treat$)).ti,ab. or (behavio$ adj3 (intervention$ or psychotherapy$ or technique$ or therap$ or treat$)).ti,ab.	189,712
97	[that came from here: James, A. C., James, G., Cowdrey, F. A., Soler, A., & Choke, A. (2015). Cognitive behavioural therapy for anxiety disorders in children and adolescents. Cochrane Database of Systematic Reviews, 2. https://doi.org/10.1002/14651858.CD004690.pub4]	0
98	86 and 96 and 92	381
99	86 and (89 or 96) and 92	393

Any amendments to the inclusion or exclusion criteria, databases, or search parameters will be documented and justified in the final manuscript, along with an assessment of their potential impact on the review findings.

### Data management

All search results from the selected databases will be imported into Zotero 7.0.26 for reference organization. The reference list will then be uploaded to Covidence [20] to facilitate title-abstract and full-text screening by the review team.

### Study selection

Two reviewers (J.D.C. and Y.Y.) will independently screen studies. First, titles and abstracts will be assessed against the predefined eligibility criteria. Subsequently, the same reviewers will evaluate the full texts of potentially eligible articles to determine final inclusion. All screening results will be cross-checked for consistency. Any disagreements between reviewers will be resolved through discussion or, if necessary, by consulting a third senior reviewer. When essential information is missing or unclear, the corresponding author of the study or the principal investigator llisted in the trial registration will be contacted for clarification. Cohen’s kappa statistic will be used to assess inter-rater agreement. A list of excluded studies and corresponding reasons for exclusion will be provided in the final review.

### Data extraction

Two reviewers (J.D.C. and Y.Y.) will extract data independently from each included study using a standardized and pre-piloted data extraction form developed in Microsoft Excel (Microsoft Corporation, Redmond, WA, USA). The form will be pilot-tested on a small subset of eligible studies to ensure clarity and consistency. Disagreements between reviewers will be resolved through discussion or consultation with a third reviewer. The inter-rater reliability for data extraction will be assessed using Cohen’s kappa statistic and will be reported.

The extraction form will include study-level and intervention-level variables, including study identification details (authors, publication year, journal, country, funding source, study registration), study characteristics (design, setting, sample size, aims, inclusion/exclusion criteria), participant characteristics (age, gender identity, sexual orientation, mental health condition, comorbidities, baseline severity, psychotropic medication use), intervention characteristics (the type of CBT intervention, duration, number and length of sessions, delivery modality, setting, therapist qualifications, theoretical adaptations), comparator details (type, duration, delivery), and outcomes assessed (depression, anxiety, PTSD, OCD, substance use disorders).

When relevant information is missing or unclear, study authors will be contacted for clarification.

### Risk of bias assessment

The methodological quality of all included RCTs will be assessed independently by two reviewers (J.D.C. and Y.Y.) using the Cochrane Risk of Bias 2 (RoB 2) tool [21]. The assessment will evaluate five domains: (1) bias arising from the randomization process, (2) bias due to deviations from intended interventions, (3) bias due to missing outcome data, (4) bias in measurement of the outcome, and (5) bias in selection of the reported result. Each domain will be rated as low risk, some concerns, or high risk of bias according to Cochrane guidelines. For completeness of outcome data (attrition bias), we will consider both the amount and balance of missing data. Consistent with Cochrane guidance,  > 20% overall attrition or >10% differential attrition will be judged as high risk. We will also note whether studies used intention-to-treat (ITT) analysis, as appropriate handling of missing data lowers the risk of attrition bias.

Discrepancies between reviewers will be resolved through discussion or consultation with a third senior reviewer. Following individual domain ratings, an overall risk of bias judgment will be assigned to each study using RoB 2’s algorithm. A summary of risk of bias for each domain and across studies will be presented both in tabular form and as visual figures (e.g., risk of bias summary plots) generated using RevMan 5.4 [22] or the robvis package [23] in R.

### Data synthesis

Data from included studies will first be summarized narratively, accompanied by descriptive tables detailing study characteristics (e.g., population/diagnosis, baseline severity, setting), intervention components (e.g., core CBT techniques, adjunct elements, delivery modality, cultural/theoretical adaptations), participant demographics, and outcomes (e.g., instrument and timepoint). When sufficient homogeneous data are available, a quantitative synthesis (meta-analysis) will be performed using the R statistical software (version 4.4.0; R Core Team, 2024). Sufficient homogeneous data will be defined as data from at least two studies with the same comparison and comparable outcomes, with I^2^ less than or equal to 50%, indicating low to moderate heterogeneity to compute an effect size.

For continuous outcomes in the meta-analysis, results will be pooled as standardized mean differences (SMDs; Cohen’s d) [24] with corresponding 95% confidence intervals (CIs). For dichotomous outcomes, risk ratios (RRs) [25] with 95% CIs will be computed. All pooled estimates will be calculated using a random-effects model [26].

We will also employ the GRADE (Grading of Recommendations, Assessment, Development and Evaluation) framework to assess the strength of evidence [27]. GRADE rates the certainty of evidence for each critical outcome as high, moderate, low, or very low by considering domains such as risk of bias, inconsistency, indirectness, imprecision, and publication bias. We may summarize judgments in a Summary of Findings table.

### Assessment of heterogeneity

Heterogeneity across studies will be assessed using Cochran’s Q test (χ²) and the I² statistic, with thresholds of 25%, 50%, and 75% representing low, moderate, and high heterogeneity, respectively. When high heterogeneity (I² ≥ 75%) is detected [28], potential sources will be explored through subgroup analyses and meta-regression, examining factors such as intervention modality (e.g., in-person vs. telehealth), target disorder, study location, and population subgroup (e.g., cisgender vs. mixed samples) when feasible.

### Publication bias analyses

To minimize potential publication bias, a comprehensive and systematic literature search will be undertaken. To further ensure the transparency and objectivity of the review, information regarding study funding sources and author conflicts of interest will be extracted and examined. When appropriate, potential publication bias will be evaluated using funnel plots [29].

### Sensitivity analysis

Sensitivity analyses will be performed by excluding studies with high risk of bias or small sample sizes to test the robustness of the pooled results. We will define “small sample” a priori as trials with fewer than 25 participants per arm, consistent with meta-epidemiological evidence that very small trials are more susceptible to small-study effects and may yield more favorable treatment estimates than larger trials [30].

## Discussion

Despite growing recognition of minority stress as a key contributor to LGBTQ mental health disparities, evidence on the effectiveness of LGBTQ-affirmative CBT remains limited and mixed. Existing trials vary widely in intervention structure, adaptation methods, follow-up duration, and outcome measurement, leading to inconsistent findings regarding efficacy. This methodological diversity makes it difficult to identify the active components driving improvement.

The present systematic review will address these gaps by synthesizing RCTs of LGBTQ adapted CBT interventions for adults. By examining short- and long-term effects across mental health outcomes and evaluating moderators such as delivery mode and intervention duration, the review aims to clarify both the effectiveness and mechanisms of affirmative CBT. The findings will provide an evidence base to inform culturally responsive clinical practice and guide future development of LGBTQ inclusive mental health treatments.

## Supporting information

S1 Appendix PRISMA-P 2015 Checklist. (DOCX)
